# Paternal Age Matters: Association with Sperm Criteria’s- Spermatozoa DNA Integrity and Methylation Profile

**DOI:** 10.3390/jcm12154928

**Published:** 2023-07-27

**Authors:** Marwa Lahimer, Debbie Montjean, Rosalie Cabry, Severine Capelle, Elodie Lefranc, Véronique Bach, Mounir Ajina, Habib Ben Ali, Hafida Khorsi-Cauet, Moncef Benkhalifa

**Affiliations:** 1 Reproductive Medicine, Reproductive Biology & Genetics, University Hospital and School of Medicine, Picardie University Jules Verne, CHU Sud, 80054 Amiens, France; marwa.lahimer@etud.u-picardie.fr (M.L.); cabry.rosalie@chu-amiens.fr (R.C.); capelle.severine@chu-amiens.fr (S.C.); lefranc.elodie@chu-amiens.fr (E.L.); benkhalifa.moncef@chu-amiens.fr (M.B.); 2 PERITOX—Périnatalité et Risques Toxiques—UMR-I1, Picardie University Jules Verne, CHU Sud, 80025 Amiens, France; veronique.bach@u-picardie.fr; 3 Exercise Physiology and Physiopathology: From Integrated to Molecular “Biology, Medicine and Health” (Code: LR19ES09), Sousse 4002, Tunisia; 4 Fertilys, Centres de Fertilité, Laval and Brossard, Québec, QC H7S 1Z5, Canada; debbie.montjean@fertilys.org; 5 Unit of Reproductive Medicine, University Hospital Farhat Hached, Sousse 4000, Tunisia; mounir.ajina@rns.tn; 6 Faculty of Medicine of Sousse, University of Sousse, Sousse 4000, Tunisia; habib.benali@famso.u-sousse.tn

**Keywords:** paternal age, sperm parameters, spermatozoa DNA integrity and methylation

## Abstract

Advanced age has been reported to negatively affect sperm parameters and spermatozoa DNA integrity. A decline in sperm criteria was also associated with altered epigenetic marks such as DNA methylation with a potential downstream impact on in vitro fertilization success and clinical outcomes. The aim of the present retrospective study was to clarify the association between advanced paternal age (APA) and sperm parameters, DNA integrity and DNA methylation profile. A total of 671 patients consulting for infertility underwent sperm analysis, sperm DNA integrity assessment and methylation level measurement. The principal finding was that individuals over 40 years of age exhibit a significant increase in DNA fragmentation levels compared to the younger group (15% versus 9%, respectively, *p* = 0.04). However, there was no significant difference in DNA decondensation and sperm parameters in association with APA. In addition, a drop in the global methylation level was also found in men over 40 years (6% in the young group versus 2% in the old group, *p* = 0.03). As a conclusion, men over 40 years are at higher risk of elevated sperm DNA fragmentation and lower methylation level. Based on these observations, it is recommended that the assessment of sperm DNA fragmentation should be taken into consideration particularly after the age of 40. Our findings support the idea that paternal age is a crucial factor that should not be neglected during fertility evaluation and treatment since it is associated with epigenetics changes in sperm. Although the underlying mechanism remains to be clarified, we believe that environmental and professional exposure factors are likely involved in the process.

## 1. Introduction

A significant decline in semen characteristics has been reported during the past decades [1,2,3,4]. Multiple factors can account for global male fertility decline such as professional occupation, endocrine disruptor exposure and advanced age [5,6]. Noteworthy, changes in semen parameters like volume, total sperm count and vitality have been associated with advanced age [7,8]. It is commonly recognized that both men and women experience changes in fertility and reproductive health as they age. While much attention has been given to the impact of maternal age on fertility and pregnancy outcomes, research has also shown that advanced paternal age can have implications for fertility, as well as the health of offspring. Evidence reported that advanced paternal age has been associated with a decline in sperm quality, including decreased sperm count, motility, and morphology. A retrospective study published by Gao in 2021 investigated a total of 18,441 samples from men aged 17–71 years. They reported that up to 30 years old, there was a decrease in viability, and up to 35 years old, sperm volume and total of sperm count, motility and sperm morphology began to decline [9]. This decline in sperm parameters can reduce the chances of successful fertilization [5]. Therefore, the analysis of conventional semen parameters provides important information about sperm quality. However, these parameters alone may not be sufficient to establish a direct and conclusive association between age and reproductive potential. A more comprehensive approach, including molecular analyses and consideration of other relevant factors, is needed to better understand the impact of age on male reproductive health and fertility.

Furthermore, age-related changes in the testes are well documented [10,11]. For example, it was reported that the number of Leydig cells may decrease with age, leading to lower testosterone levels and reduced number of type A spermatogonia. Conversely, some studies showed no association of advanced paternal age (APA) over 40 years with conventional semen parameters. However, a negative correlation was found between advanced paternal age (APA) and sperm DNA integrity, embryonic quality, and clinical IVF outcomes. The study observed that APA was associated with a reduction in the rate of expanded blastocyst formation and an increased risk of miscarriage, particularly when DNA fragmentation exceeded 20% [5,12,13,14,15,16,17].

Although the latest data about the impact of APA on reproductive outcome are conflicting, there is a growing body of evidence that supports the fact that APA may contribute not only to lower semen quality but also to offspring vulnerability to inheritable diseases [18,19]. Indeed, genetic abnormalities, such as chromosomal aneuploidy, DNA mutations, epigenetic modifications, and gene silencing have been associated with APA which in turn may favorize premature birth and the onset of other diseases, including autism, schizophrenia, bipolar disorders, and pediatric leukemia [5,20,21]. Epidemiologic studies have supported that APA can impact the genetic and epigenetic status of the spermatozoa causing idiopathic male infertility and poorer IVF outcomes [22,23]. DNA methylation is one of epigenetic modifications that can affect gene expression without changing the DNA sequence [24], and plays a crucial role in spermatogenesis [25]. A number of studies have investigated the implication of sperm DNA methylation on male infertility [26,27,28]. Research in human and animal models suggests that advanced paternal age can lead to alterations in sperm DNA methylation [29,30]. These changes can occur in specific genes or regions of the genome and may be associated with semen quality decline and affect the potential of fertility [31]. Overall, data on the association of APA with sperm decay and sperm DNA methylation remain limited and inconclusive.

Nowadays, couples seeking assisted reproductive techniques, such as in vitro fertilization (IVF) or intracytoplasmic sperm injection (ICSI), may face challenges due to advanced paternal age. However, the assisted reproductive techniques can improve the chances of conception, but they may not completely eliminate the risks of genetic abnormalities associated with advanced paternal age.

The present study was designed with the specific objective of investigating and challenging the association between advanced paternal age (APA) and various aspects of male reproductive health, including sperm parameters, sperm DNA integrity, and sperm methylation profile. Focusing on these specific areas, this research aimed to provide a comprehensive understanding of the impact of advanced paternal age on male fertility and reproductive health. The study sought to challenge the existing knowledge and explore any potential inconsistencies or gaps in the current understanding of the association between APA and these reproductive parameters.

## 2. Materials and Methods

### 2.1. Study Participants

In this prospective study, data from a total of 671 patients who sought male fertility investigation at the Reproductive Medicine Unit of the University Hospital of Amiens (Picardie University Jules Verne) were evaluated. It is important to note that there were no specific selection criteria for the inclusion of patients in this study, ensuring a diverse representation of individuals seeking fertility assessment. The study population was divided into two distinct groups for comparative analysis. The first group, referred to as G1, consisted of men under the age of 40. The second group, known as G2, included men who were 40 years or older than 40. This age-based division allowed comparison of fertility-related parameters between the two age groups.

### 2.2. Semen Analysis

Fresh semen samples were meticulously collected through masturbation, ensuring a sterile container was used, following a recommended sexual abstinence period of 3 to 5 days. After a period of 30 min of liquefaction at 37 degrees Celsius, each sample underwent a comprehensive evaluation. Various parameters were assessed, including pH level (providing valuable insights into the overall health of the semen), viscosity (or consistency which can offer indications about the presence of any abnormalities or potential fertility issues), color (which can sometimes indicate underlying health conditions), volume (to determine the quantity of semen produced during ejaculation), and several sperm-related parameters.

### 2.3. Sperm Preparation

Sperm were treated with a discontinuous PureSperm^®^ gradient (Nicadon, Mölndal, Sweden) consisting of a lower 80% layer (2.4 mL of PureSperm^®^ reagent + 0.6 mL of HEPES) and an upper 40% layer (1 mL of PureSperm^®^ reagent + 1 mL of the 80% layer). Perhaps 1 mL of semen sample (depending on the sperm count and sample volume) was added to the 40% layer. After centrifugation at 300× *g* for 20 min, the supernatant was discarded, and the 80% layer was collected then washed with 1 mL of Ferticult Flushing medium (FertiPro N.V., Beernem, Belgium). After centrifugation at 600× *g* for 10 min, the final pellet (containing total migrated spermatozoa) was washed twice in 1 mL of phosphate-buffered saline (PBS) and centrifuged at 600× *g* for 10 min.

### 2.4. Sperm Parameter Assessment

The conventional sperm parameters were assessed according to the classification of the World Health Organization (WHO)’s Laboratory Manual for the Examination of Human Semen and Sperm-Cervical Mucus Interaction, sixth edition (2010/2021) [32].

- The sperm count was calculated using a 10-chambered hemocytometer-type grid (Glasstic™ Slide 10 with Grids, Kova International Inc., Garden Grove, CA, USA).
- Sperm vitality was assessed using an eosin/nigrosine stain that colored dead spermatozoa pink. The percentage of vitality is calculated as the non-colored spermatozoa out of a total of 100 spermatozoa.
- Sperm motility is defined as the percentages of progressive spermatozoa (type a), non-progressive spermatozoa (type b), slow spermatozoa (type C), and non-mobile spermatozoa (type D). The motility was evaluated using a light microscopy (magnification: ×40). In the present study, motility was expressed as the percentage of progressive and non-progressive spermatozoa type (a + b).
- According to Kruger’s classification in the WHO 2010/2021 guidelines, the teratozoospermia is defined as when the percentage of atypical forms of spermatozoa is more than 4%. The sperm morphology was evaluated using Shorr staining. A spermatozoon presenting at least one of the following features was classified as atypical or abnormal: macrocephaly, microcephaly, a tapered head, globozoospermia, lack of a head, lack of a tail, and a coiled tail. The percentage of atypical forms was recorded in a total of 100 spermatozoa.

This comprehensive analysis helps in diagnosing potential fertility issues, determining appropriate treatment plans, and guiding decisions related to assisted reproductive techniques.

### 2.5. Sperm DNA Fragmentation (DFI) Analysis: Tunel Assay

In addition to the parameters mentioned earlier, the DNA fragmentation index (DFI) is another crucial factor assessed during semen analysis. The DFI represents the percentage of sperm cells within a sample that have fragmented DNA. DNA fragmentation refers to the breakage or damage to the genetic material within the sperm cells.

Part of the semen sample was incubated with Triton^TM^ X-0.1% (Sigma, Darmstadt, Germany) for 5 min at 4 °C and smeared on a slide followed by a 3-h incubation in glutaraldehyde 3% PBS for fixation. After 3 steps of washing in PBS (phosphate-buffered saline), we applicate the tunel kit [terminal deoxynucleotidyl transferase (TdT)-mediated dUDP nick-end labelling], fluorescein (ROCHE). In addition, we added a positive control and a negative control. These controls serve as reference points for comparison.

- The positive control refers to a slide that is treated with DNase I, an enzyme that breaks down DNA. The slide is incubated with DNase I at a concentration of 1000 U/mL for 15 min at a temperature of 37 degrees Celsius.
- The negative control refers to a slide that is treated with PBS (phosphate-buffered saline), a neutral solution without any active substances. The slide is incubated with PBS alone, without any additional treatments.

The slides were revealed using DAPI (4′,6-diamidino-2-phenylindole) and read under fluorescence microscopy. Sperm fragmentation was calculated as the percentage of spermatozoa showing fluorescence out of a total of 200 spermatozoa.

To establish a reference point for evaluating DFI, a threshold value is set. In this case, the threshold for DFI is defined as up to 30%. This means that if the percentage of sperm with fragmented DNA exceeds 30% in a given sample, it is considered to be beyond the normal range [33].

### 2.6. Sperm DNA Decondensation Index SDI

In order to evaluate the quality and maturity of sperm cells, we realized a specific staining technique involving aniline blue and acetic acid to assess the degree of compaction or packaging of DNA molecules within the sperm cells. The sperm cells were initially washed in phosphate-buffered saline (PBS) to remove any impurities. Subsequently, the cells were fixed using a solution of 3% glyceraldehyde in PBS. To ensure accuracy and reliability, all slides were prepared in duplicate. The fixed sperm cells were incubated in the mixture of aniline blue and acetic acid for a duration of 10 min at room temperature. During this incubation period, the aniline blue dye penetrates the sperm cells and binds to DNA molecules. This staining method allows the visualization of sperm chromatin condensation levels. The sperm chromatin de-condensation index (SDI) was calculated by counting the total number of spermatozoa that exhibited aniline blue staining. The intensity of aniline blue staining is proportional to the level of DNA condensation. This counting process was performed on 200 spermatozoa from each sample. By quantifying the number of aniline-blue-stained spermatozoa, the SDI provides an indication of the degree of DNA condensation within the sperm cells [34].

### 2.7. Sperm DNA Methylation Profile Analysis: Immunoassay

In this study, an immunoassay technique was employed to examine the methylation profile of sperm DNA, specifically targeting the 5-methylcytosine (5-mc) DNA base. A mouse antibody (AC N° ab.73938, Abcam, Cambridge, UK) designed to bind to 5-mC was utilized as the primary antibody. The primary antibody was allowed to incubate overnight at 4 °C which provokes a specific binding of the antibody to the 5-mC sites within the sperm DNA. Following the primary antibody incubation, the slides were subjected to a one-hour incubation at room temperature with a secondary antibody labeled with FITC (AC N° ab8517, Abcam, Cambridge, UK), which specifically recognizes the primary mouse antibody.

To visualize the spermatozoa nuclei, a counterstaining technique using DAPI was employed. DAPI is a fluorescent stain that selectively binds to DNA, emitting a blue fluorescence signal. To assess the level of sperm DNA methylation, a specific number of spermatozoa (200 spermatozoa) were examined under a fluorescence microscope. The sperm cells that exhibited green fluorescence, indicating the presence of 5-mC, were counted. By calculating the percentage of green-stained spermatozoa out of the total number examined (200), the level of sperm DNA methylation can be determined. This immunoassay technique was evaluated in a cohort of 200 patients, providing valuable insights into their sperm DNA methylation patterns.

### 2.8. Statistical Data Analysis

SPSS 22 (SPSS Inc., Chicago, IL, USA) was used to analyze the collected data. The results were presented as mean values ± standard deviations (SD) or median [minimum–maximum] as appropriate. We compared the group G1 (men under 40 years of age) and the group G2 (men of or over 40 years of age). Continuous variables were examined for normality using the Kolmogorov–Smirnov test. t-Student test was used to analyze the quantitative independent parameters, and the Mann–Whitney Wilcoxon test was used to analyze the non-parametric data. Statistical differences were considered as significant where *p* < 0.05. Volume and body mass index (BMI) were analyzed as categorical variables. Other sperm parameters including DNA integrity were analyzed as continuous variables.

## 3. Results

In this study, a total of 671 semen samples were analyzed, as indicated in Table 1. The cohort of individuals included in the analysis had a mean paternal age of 34.3 years, with a standard deviation (SD) of 6.5 years. The age range of the participants varied from 20 to 57 years. The mean body mass index (BMI) of the study population was calculated to be 26.41 kg/m^2^, with a standard deviation of 4.9 kg/m^2^. The range of BMI values observed within the cohort ranged from 16.4 kg/m^2^ to 51.6 kg/m^2^. These measurements provide insights into the body composition and weight status of the individuals included in the study.

**Figure 1:**
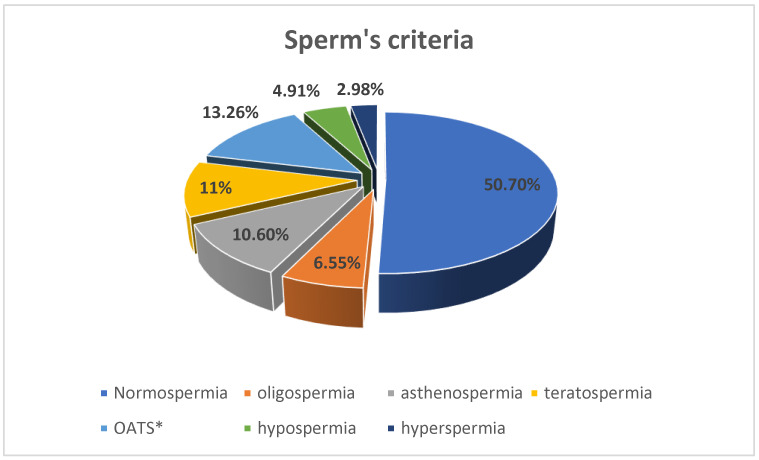
Patients’ distribution according to conventional sperm parameters (The Sixth Edition of the WHO Manual for Human Semen Analysis 2021, 5th centile). Normospermic subjects: sperm concentration 16 × 106/mL; total motility ≥ 42%; typical forms ≥ 4%; viability ≥ 58%; volume ≥ 1.4 mL. Oligozoospermia (sperm concentration < 16 M/mL); Asthenozoospermia (% motility < 42%), Teratozoospermia (typical form < 4%); Oligoasthenoteratozoospermia (OATS) subjects: sperm concentration < 16 M/mL, total motility < 42%, typical form < 4%); reduced volume (hypo-spermia) < 1.4 mL). Increased volume (hyper-spermia) (95% confidence) volume > 6.2 mL). *: Oligoasthenoteratozoospermia [35].

According to the World Health Organization (WHO) criteria from 2021, approximately 50.7% of the studied population are classified as normospermic. This information is depicted in Figure 1, which provides a visual representation of the distribution of patients based on their sperm parameters. The normospermic subjects are considered as subjects with normal sperm parameters but they can be infertile subjects. The details of distribution in the other categories (oligospermia, asthenospermia, teratospermia, hypospermia, hyperspermia and asthenospermia) were assessed to fully understand the overall characteristics of the studied population and their semen analysis results.

Based on paternal age, the studied population was divided into two distinct groups for comparative analysis. G1 (men under the age of 40 years). The second group, G2 (included men aged equal to or older than 40 years). The division into these groups allowed for the examination of the impact of advanced paternal age (APA) on the various parameters.

The conventional semen analysis conducted in the study did not reveal a significant association between sperm parameters, including volume, concentration, motility, viability, morphology, and advanced paternal age (APA). The comparison between Group 1 (G1) and Group 2 (G2) did not show any significant differences in these parameters, indicating that paternal age did not have a substantial impact on these specific sperm characteristics (Table 1).

**Figure 2:**
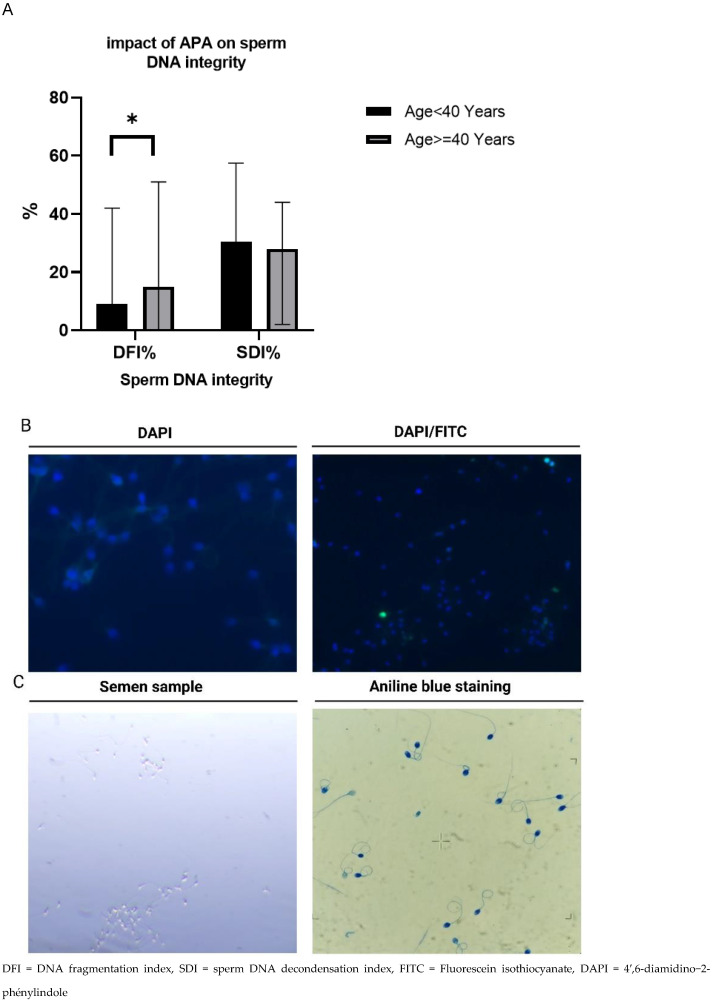
Impact of advanced paternal age on sperm DNA integrity. (**A**) DFI was relatively high in G1 compared to G2, * *p* < 0.05, no significant difference in SDI between the two groups. DNA fragmentation refers to the physical breakage or damage to the DNA strands within sperm cells. (**B**) Detection of single- or double-strand breaks in sperm DNA. The fragmented sperm DNA is visualized as a green color conversely, non-fragmented sperm DNA is represented by a blue color using the DAPI dye. (**C**) Microscopic observation of the aniline blue staining, the blue color indicates the penetration of aniline blue into decondensed chromatin within the spermatozoa. It represents an indicator of the spermatozoa’s maturity.

The analysis of sperm DNA integrity data shows a significant difference in sperm DNA fragmentation levels between (G1) and (G2) based on paternal age (threshold of 40 years). Specifically, G2 exhibited a higher level of DNA fragmentation compared to G1. The DNA fragmentation level in G2 was measured at 15%, while G1 demonstrated a lower level of 9%. This difference was statistically significant, as indicated by a *p*-value of 0.04, suggesting a meaningful distinction between the two groups (Figure 2A,B). On the other hand, the analysis revealed that the sperm DNA decondensation index within sperm cells did not show a significant difference between G1 and G2. This suggests that the level of DNA decondensation, an indicator for spermatozoa maturity, is not affected by advanced paternal age (Figure 2A,C).

These findings indicate that advanced paternal age is associated with an increased risk of DNA fragmentation in sperm cells. The higher DNA fragmentation levels observed in G2 compared to G1 suggest that paternal age may be a contributing factor to DNA damage in sperm, potentially impacting fertility outcomes.

The assessment of methylation levels in the studied population revealed a significant decrease in methylation with aging, specifically in relation to advanced paternal age (APA). (G2) exhibited a lower methylation level compared to (G1).

**Figure 3:**
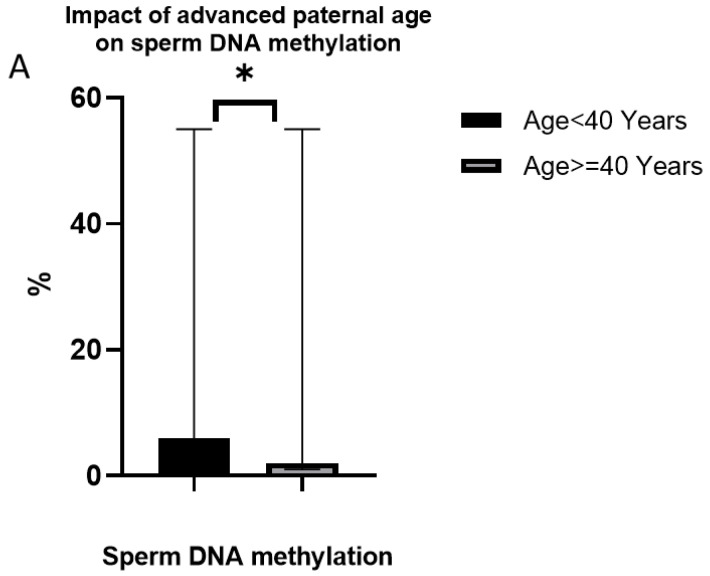

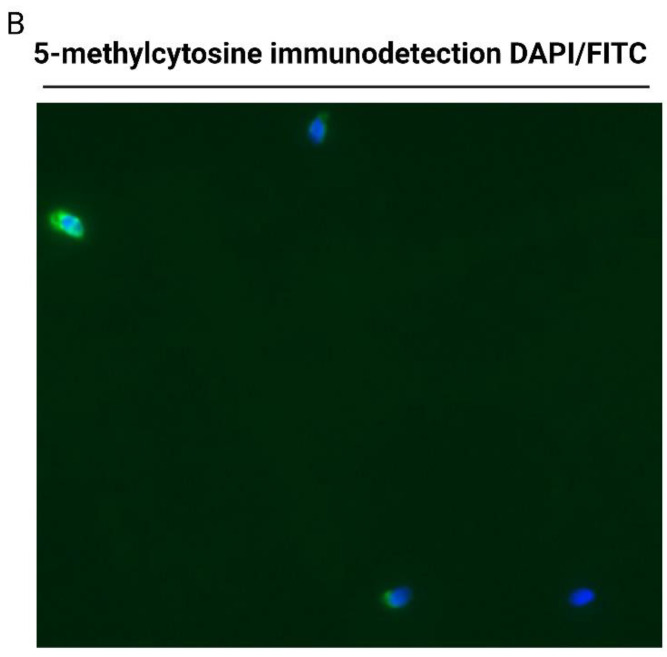
Impact of advanced paternal age on DNA methylation profile. (**A**) Decrease in sperm DNA methylation levels in G2 compared to G1, * *p* < 0.05. (**B**) Detection of 5′-methylcytosine in spermatozoa, the binding of the anti-5-methylcytosine antibody is denoted by the green color, while the absence of binding is visualized as a blue color using the DAPI dye.

The methylation level in G2 was measured at 2.0%, while G1 demonstrated a higher level of 6.0%. This difference was found to be statistically significant, as indicated by a *p*-value of less than 0.05. These findings suggest that advanced paternal age is associated with a decline in methylation levels in sperm cells (Figure 3A,B). The observed decrease in methylation levels with aging highlights the potential impact of epigenetic changes on male reproductive health. This finding suggests that advanced paternal age may influence the epigenetic landscape of sperm cells, which can have implications for fertility outcomes.

## 4. Discussion

Our results are in line with previous observations. Indeed, we found that conventional sperm parameters including volume, concentration, morphology, motility, and vitality were not significantly different in the advanced paternal age group. These outcomes are consistent with previous studies highlighting that conventional sperm parameters may not be greatly affected by advanced paternal age [5]. This finding suggests that advanced paternal age may not have a substantial impact on these specific sperm parameters. In contrast to our findings, a study published in 2001 showed a significant decline in sperm morphology, motility, and progressive motility with age [36]. In addition, according to a meta-analysis, one cannot exclude a possible significant decrease in sperm concentration and motility starting from 35 years old and continuing with aging [8]. This decline in semen quality is observed in both fertile and infertile men, but the effect is more pronounced in the infertile group. Furthermore, it was reported from 2681 patients that after 50 years of age the sperm parameters can be significantly affected especially the volume, the concentration, sperm DNA fragmentation and chromatin denaturation [7]. The inconsistencies in results among previous studies related to advanced paternal age and sperm parameters highlight the need for further research and standardization in defining the age groups to improve the understanding of the effects of advanced paternal age on male fertility.

We found that APA had an impact on DNA fragmentation. In fact, a detrimental effect of age has already been reported on DNA fragmentation; chromatin decondensation and even on sperm aneuploidy rates [37]. The statement suggests that advanced male age can have a negative influence on fertility potential, similar to the impact on women. This is an important finding since an increased number of couples are choosing to have children at an older age. Out of the 19 studies, 17 demonstrated a significant association between advanced paternal age and DNA fragmentation index in men similarly to previous studies [9,38,39,40]. Furthermore, a study including 25,445 men consulting for infertility revealed a dramatic increase in sperm DNA fragmentation was found in men over 40 years of age [41]. The relationship of age with epigenetic changes in spermatozoa was proven by Jenkins et al. They also associated this observation with an increased time to pregnancy and a higher susceptibility for the offspring to develop specific diseases [20]. In fact, epigenetic modifications of sperm chromatin are involved in genome reprogramming process and gene expression. Therefore, more information is needed regarding the relation of epigenetic changes in sperm and subsequent embryo development. However, epigenetic modifications in gametes and early embryogenesis are becoming an important field of investigation in assisted reproduction [31].

In our study, the analysis of sperm DNA methylation levels revealed a significant decrease or drop in methylation levels associated with advanced paternal age (APA). This finding suggests that there may be an association between APA and alterations in sperm DNA methylation patterns. Epigenetic changes at specific loci were proven to happen in men with altered spermatogenesis [5,20,21,27,28,42,43,44]. Moreover, epigenetic changes have been proposed as an outcome predictor of assisted reproductive technology treatment. For example, studies have suggested that DNA methylation patterns in the sperm may be predictive of successful fertilization and embryo development following IVF [45]. Additionally, idiopathic male infertility, which refers to infertility of unknown cause, is an area where epigenetic markers may hold promise [25]. Some studies have found differences in DNA methylation profiles in sperm from men with idiopathic infertility compared to fertile men, suggesting that epigenetic changes may play a role in this condition [26]. There is growing interest in epigenetic mechanisms through which paternal influences offspring development. Different studies reported that fathering age can be associated with different diseases such as psychiatric disorders like autism, schizophrenia, and neurodevelopmental disease. These findings suggest a transmission of epigenetics changes through the male germline [46,47,48,49].

It is interesting to note that age affects all known epigenetic mechanisms, including DNA methylation, histone and small non-coding (snc) RNA modifications. While DNA methylation is the most studied epigenetic mark, there is an ongoing debate about the tendency of age-related changes, whether it is a loss or a gain of epigenetic marks [50]. However, there is a growing body of evidence of the impact of age on gamete epigenetic landscape suggesting the existence of a “biological clock” in males similarly to females. This clock likely involves epigenetic changes, including DNA methylation. Therefore, methylation analysis may turn out to be a useful tool for predicting age-related changes in human sperm [51].

## 5. Conclusions

The integrity of sperm DNA is one of the factors associated with successful reproductive outcomes. Therefore, clinicians may consider testing for DNA fragmentation and DNA methylation in men over 40 years of age. This study supports the data about the effect of APA on both DNA fragmentation and DNA methylation. Indeed, conventional semen analysis can fail to explain male fertility issues. Further investigations and studies are necessary to comprehensively explore the potential effects of advanced paternal age on various aspects of male fertility. Additional parameters or advanced techniques, such as DNA fragmentation analysis, may provide a more comprehensive understanding of the impact of paternal age on sperm quality and fertility outcomes. Overall, our study results highlight the importance of exploring alternative methods to enhance the assessment of spermatozoa quality. Additionally, while the exact mechanism underlying methylation loss in advanced paternal age (APA) is yet to be fully elucidated, further investigation is warranted to better understand this phenomenon. The impacts of increased exposure to professional and environmental toxic factors are likely involved in male fertility declining in combination with late age fathering.

### Limitations and Perspectives

Regarding the strengths of our analysis, we followed an efficient protocol and included a large number of patients, which resulted in significant findings related to the risk of advanced paternal age. However, our study does have some limitations. For instance, we utilized a high-performance technique, specifically Illumina technology, to measure DNA methylation levels.

Moving forward, our forthcoming research aims to explore the association between advanced paternal age and embryonic quality. We also plan to raise the age threshold for study participants to 50 years old. By increasing the age threshold, we hope to assess the subsequent impact on various factors, including conventional sperm parameters, DNA fragmentation, DNA de-condensation, DNA methylation, embryonic quality, and the outcomes of in vitro fertilization (IVF).

## Figures and Tables

**Table 1:** Comparison of semen parameters between the two study groups. In the table, the mean values ± standard deviations (SD) are presented for volume and body mass index (BMI), which indicate the average values and their variability within each group. The other sperm parameters, such as sperm concentration, total motility, progressive motility, morphology, viability, and total sperm count, are presented as median values [minimum–maximum].

Sperm Parameter	G1 (Age < 40) *n* = 504	G2 (Age ≥ 40) *n* = 167	*p*-Value
BMI (kg/m^2^)	26.48 ± 5.1	26.1 ± 3.6	0.46
Volume (mL)	3.7 ± 1.7	3.4 ± 1.7	0.08
Concentration (10^6^ spz/mL) *	41.4 [0; 625.6]	30 [0; 248]	0.87
Motility (%)	50 [0; 87]	50 [0; 80]	0.90
Viability (%)	85 [31; 97]	85 [6; 92]	0.06
Typical form (%)	11 [0; 39]	12.5 [0; 36]	0.2

* Spz: spermatozoa.

## Data Availability

Not applicable.
